# *Erysiphedeutziicola* sp. nov. (Erysiphaceae, Ascomycota), a powdery mildew species found on *Deutziaparviflora* (Hydrangeaceae) with unusual appendages

**DOI:** 10.3897/mycokeys.51.34956

**Published:** 2019-05-08

**Authors:** Peng-Lei Qiu, Uwe Braun, Yu Li, Shu-Yan Liu

**Affiliations:** 1 Jilin Agricultural University, Engineering Research Center of Chinese Ministry of Education for Edible and Medicinal Fungi, No. 2888 Xincheng Street, Changchun 130118, Jilin Province, China; 2 Martin Luther University, Institute of Biology, Geobotany and Botanical Garden, Herbarium, Neuwerk 21, 06099 Halle (Saale), Germany; 3 Laboratory of Plant Pathology, College of Plant Protection, Jilin Agricultural University, No. 2888 Xincheng Street, Changchun130118, Jilin Province, China

**Keywords:** Erysiphales, powdery mildew, pathogen, ITS, 28S rDNA, phylogeny

## Abstract

A powdery mildew (Erysiphales) has recently been collected on leaves of an ornamental shrub *Deutziaparviflora* in Baihua Mountain, Beijing, China. Microscopic examination of the chasmothecia suggested a species belonging to Erysiphesect.Erysiphe, above all due to mycelioid chasmothecial appendages, although circinate apices of the appendages were rather in favour of Erysiphesect.Uncinula, which is a fairly rare combination of appendage characteristics in *Erysiphe*. Phylogenetic analyses of ITS and 28S rDNA sequences demonstrated that the two examined powdery mildew collections on *D.parviflora* clustered together as an independent lineage within *Erysiphe* with 100% bootstrap support, representing a species of its own, which is phylogenetically allied to, but clearly distinct from *Erysiphedeutziae* and, in addition, morphologically quite different from all known *Erysiphe* species on hosts belonging to the Hydrangeaceae. The new species on *D.parviflora* is described as *Erysiphedeutziicola*.

## Introduction

The family Hydrangeaceae comprises 17 genera and about 220 species distributed in temperate and subtropical regions of the Americas, Pacific islands, Asia and Europe (Kubitzki 2004). One of the largest genera, *Deutzia*, includes important ornamentals and is known to be used to treat enuresis, malaria and scabies in China (He 1990). Amongst the genera of Hydrangeaceae, *Deutzia*, *Hydrangea*, *Schizophragma*, *Jamesia* and *Philadelphus* have been reported as hosts of powdery mildews (Braun and Cook 2012). Nine species are currently known on hosts of these genera, viz., *Erysiphedeutziae* (Bunkina) U. Braun & S. Takam. on *Deutzia*, *E.hydrangeae* (Z.X. Chen & R.X. Gao) U. Braun & S. Takam. on *Hydrangea*, *E.poeltii* U. Braun on *Hydrangea*, *E.schizophragmatis* (Tanda & Y. Nomura) U. Braun & S. Takam. on *Hydrangea* and *Schizophragma*, *E.yanshanensis* T.Z. Liu & U. Braun on *Hydrangea*, *Golovinomycesorontii* on *Hydrangea*, *Phyllactiniajamesiae* U. Braun on *Jamesia*, *P.philadelphi* (Jacz.) Bunkina on *Philadelphus* and *Pseudoidiumhortensiae* (Jørst.) U. Braun & R.T.A. Cook on *Hydrangea*. *Erysiphedeutziae* has been the only powdery mildew species hitherto found on *Deutzia* spp. (Braun and Cook 2012). This species was originally described as *Microsphaeradeutziae* (Bunkina 1973). In 1977, this species was recorded from the Russian Far East and Japan (Nomura 1997). Braun and Takamatsu (2000) re-allocated *M.deutziae* to *Erysiphe* based on the phylogenetic analysis of ITS rDNA sequences (Braun and Takamatsu 2000). Later, this powdery mildew was introduced to Europe with records from France, Germany, Poland and Switzerland (Bolay et al. 2005) and the UK (Denton and Henricot 2007). In recent years, this pathogen was also reported on *Deutzia* in Korea (Park et al. 2010) and China (Nguyen et al. 2018).

In 2018, leaves of *D.parviflora* with clearly dense powdery layers were collected twice. Microscopic examination suggested the unusual appendages of chasmothecia of the fungus are apparently distinct from *E.deutziae* on *Deutzia*. In order to circumscribe this species, morphological and molecular phylogenetic analyses, based on ITS and 28S rDNA sequences, were conducted for the characterisation and identification of a new *Erysiphe* species, *E.deutziicola*, found in China on *D.parviflora*.

## Materials and methods

### Morphological studies

In May 2018, *D.parviflora* plants with typical white powdery mildew symptoms were first noticed and collected in the nature reserve of Baihua Mountain of Beijing, China (115°34.20'E; 39°50.40'N) and later, in October, the sexual morph was found. The two specimens were deposited in the Herbarium of Mycology of Jilin Agricultural University (**HMJAU**) under the accession number HMJAU-PM91860 and HMJAU-PM91861, respectively. The dried specimens were put in lactic acid for light microscopic examinations (Zeiss Axio Scope A1, Germany).

### DNA extraction and sequencing

Genome DNA was extracted using chasmothecia of HMJAU-PM91860 and conidia and mycelia from the asexual specimen HMJAU-PM91861 by the Chelex-100 method (Walsh et al. 1991; Hirata and Takamatsu 1996). Two specimens of *Erysiphedeutziae* on Deutziaparvifloravar.amurensis (Nguyen et al. 2018) were also used for the DNA extraction and amplification, since 28S rDNA sequences of *E.deutziae* were not yet available in GenBank. The DNA amplification and sequencing were conducted according to the procedure described by Qiu et al. (2018).

### Molecular phylogenetic analyses

The newly obtained sequence data (28S rDNA, including domains D1 and D2 and ITS, including the 5.8S rDNA) from two powdery mildew specimens on *D.parviflora* were aligned to confirm the homology. The new sequences were deposited in GenBank under accession numbers MK656288 (ITS) and MK656309 (28S) from HMJAU-PM91860 and MK656289 (ITS) and MK656310 (28S) from HMJAU-PM91861. The combined datasets of ITS and 28S rDNA sequences from the two specimens were aligned with closely related sequences of *Erysiphe* spp. retrieved from GenBank (Table 1) including sequences from some species occurring on hosts belonging to the Hydrangeaceae using MUSCLE implemented in the MEGA 7 programme (Kumar et al. 2016). Alignments were further manually refined and deposited in TreeBASE (http://www.treebase.org/) under the accession number of S24214.

**Table 1.** Vouchers, hosts and GenBank accession numbers of the sequences used in this study.

**Species**	**Voucher**	**Host**	**Host family**	**Accession number**	**Sequence size (bp)**	**Reference**
* Erysiphe adunca *	MUMH 171	* Salix futura *	Salicaceae	LC028968	1326	Takamatsu et al. 2015b
* E. arcuata *	MUMH 2741	* Carpinus tschonoskii *	Betulaceae	AB252473	1335	Braun et al. 2006
* E. arcuata *	MUMH 3620	* C. tschonoskii *	Betulaceae	AB252474	1335	Braun et al. 2006
* E. blasti *	MUMH 0002	* Laurus umbellata *	Lauraceae	LC009905	1317	Takamatsu et al. 2015a
* E. deutziae *	HMJAU91777	Deutzia parviflora var. amurensis	Hydrangeaceae	MH027420 (ITS)	671	Nguyen et al. 2018
				MK656311 (28S)	637	This study
* E. deutziae *	HMJAU91771	D. parviflora var. amurensis	Hydrangeaceae	MG674082 (ITS)	670	Nguyen et al. 2018
				MK656312 (28S)	637	This study
* E. deutziicola *	HMJAU-PM91860	* D. parviflora *	Hydrangeaceae	MK656288 (ITS)	666	**This study**
				MK656309 (28S)	636	
* E. deutziicola *	HMJAU-PM91861	* D. parviflora *	Hydrangeaceae	MK656289 (ITS)	666	**This study**
				MK656310 (28S)	636	
* E. epigena *	MUMH 2193	* Quercus variabilis *	Fagaceae	AB292720	1403	Takamatsu et al. 2007
* E. heraclei *	MUMH 2484	* Conium maculatum *	Umbelliferae	LC010021	1355	Takamatsu et al. 2015a
* E. huayinensis *	MUMH 4644	* Isodon umbrosus *	Lamiaceae	LC010072	1314	Takamatsu et al. 2015a
* E. huayinensis *	MUMH 0087	* I. trichocarpus *	Lamiaceae	LC010080	1362	Takamatsu et al. 2015a
* E. hydrangeae *	MUMH 0514	* Hydrangea paniculata *	Hydrangeaceae	LC028983	1361	Takamatsu et al. 2015b
* E. izuensis *	MUMH 4651	* Rhododendron reticulatum *	Ericaceae	LC010076	1350	Takamatsu et al. 2015a
* E. juglandis *	TPU 1745	* Pterocarya rhoifolia *	Juglandaceae	LC010090	1276	Takamatsu et al. 2015a
* E. pileae *	MUMH 2987	* Pilea pumila *	Urticaceae	LC010059 (ITS)	552	Takamatsu et al. 2015a
				LC010058 (28S)	754	
* E. pedaliacearum *	MUMH 412	* Sesamum indicum *	Pedaliaceae	LC342968	1516	Shin et al. 2018
* E. phyllanthi *	MUMH 0099	* Phyllanthus flexuosus *	Euphorbiaceae	LC009921	1351	Takamatsu et al. 2015a
* E. sedi *	MUMH 2576	*Sedum* sp.	Crassulaceae	LC010046	1321	
* E. schizophragmatis *	MUMH 4642	* Hydangea petiolaris *	Hydrangeaceae	LC029001	1356	Takamatsu et al. 2015b
* Pseudoidium hortensiae *	MUMH 0071	* Hydrangea macrophylla *	Hydrangeaceae	LC009915	1249	Takamatsu et al. 2015a
* Pse. neolycopersici *	MUMH 0066	* Lycopersicon esculentum *	Solanaceae	LC009912	1344	Takamatsu et al. 2015a

A phylogenetic tree was obtained from the combined data using the maximum-parsimony (MP) method in PAUP 4.0. The MP analysis was performed with the heuristic search option using the tree-bisection-reconstruction (TBR) algorithm with 100 random sequence additions to find the global optimum tree. The gaps were treated as missing data. The bootstrap analysis (1000 replications) was used for testing the strength of the internal branches of the resulting trees (Felsenstein 1985). Tree scores, including tree length, consistency index (CI), retention index (RI) and rescaled consistency index (RC) were also calculated. Bootstrap (BS) values of 60% or higher are indicated.

## Results

### Phylogenetic analysis

**Figure 1.**
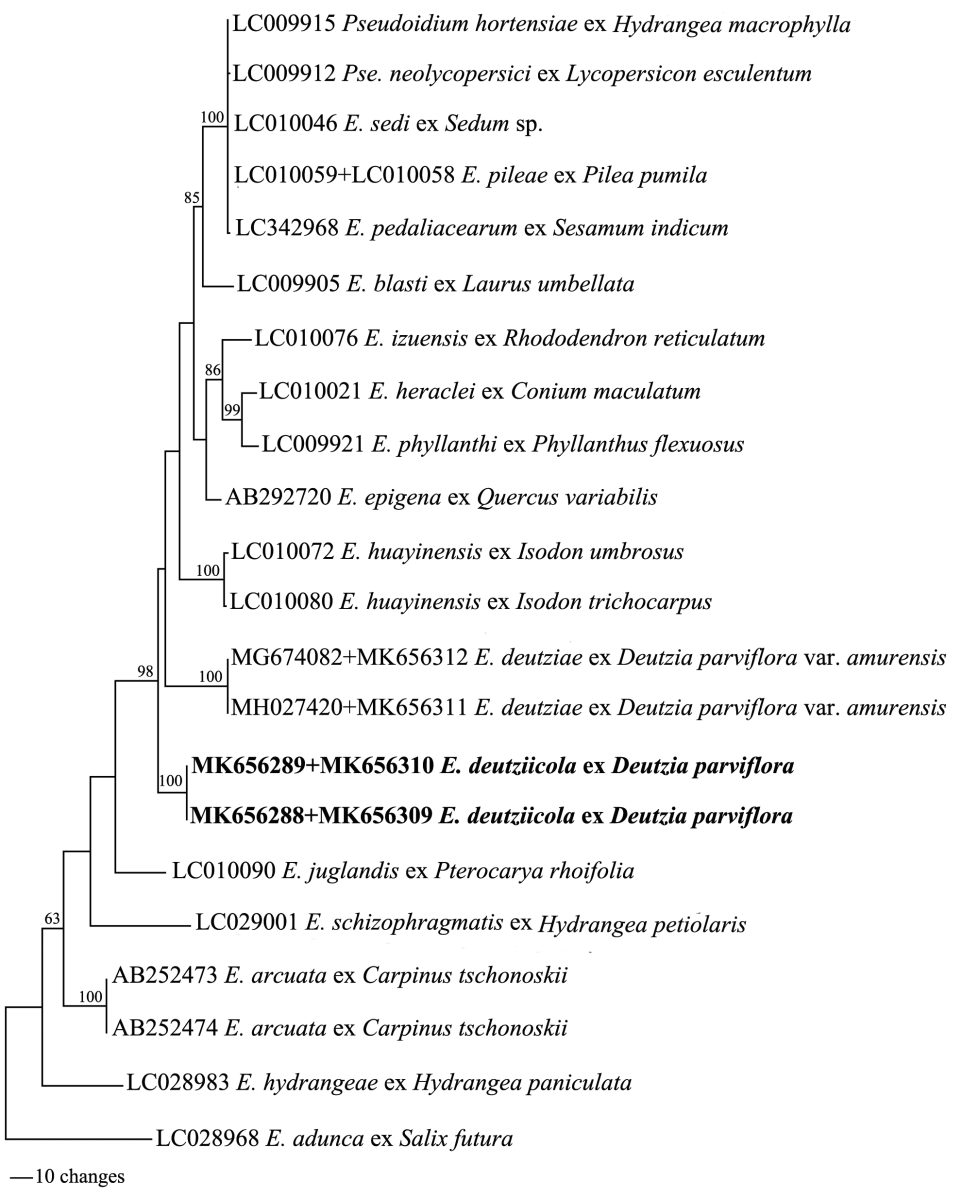
Maximum parsimony phylogram of *Erysiphedeutziicola* and its allied species constructed from the combination of ITS and 28S rDNA sequences. *Erysipheadunca* (LC028968) was used as outgroup. Bootstrap values (> 60%) by the maximum parsimony (MP) method are shown on the respective branches. The sequences pertaining to *E.deutziicola* are shown in bold face.

The alignments of ITS and 28S rDNA sequences obtained from the two specimens examined are identical to each other. A total of 22 combined sequence data, including sequences from *Pseudoidiumhortensiae*, *E.hydrangeae*, *E.schizophragmatis* and *E.deutziae*, four powdery mildew species on hosts of the Hydrangeaceae, were used to construct the phylogenetic tree. The sequence of *E.adunca* (LC028968) was used as outgroup. The original alignment dataset comprises of 1232 characters. We manually deleted 111 characters and the remaining 1121 characters were finally used for constructing the phylogenetic tree, where 105 characters were variable but not informative and 175 characters were phylogenetically informative for parsimony analysis. The analysis produced three equally parsimonious trees. The best MP tree (TL = 525, CI = 0.6895, RI = 0.7175, RC = 0.4947) with the highest likelihood score is shown in Figure 1. The phylogenetic analysis confirmed that the new sequences obtained from the powdery mildew on *D.parviflora* formed an independent clade supported by a bootstrap value of 100%.

## Taxonomy

### Erysiphe deutziicola

Taxon classificationFungiErysiphalesErysiphaceae

P.-L. Qiu, S.-Y. Liu & Y. Li sp. nov.

830253

Figure 2

#### Etymology.

Named after the host genus, *Deutzia*, + -icola (dweller).

#### Diagnosis.

Differs from all known *Erysiphe* species on hosts belonging to the Hydrangeaceae in having very long conidiophores, up to 235.0 µm and chasmothecia with mycelioid appendages, circinate at the apex.

#### Type.

CHINA. Beijing City, Baihua Mountain, on leaves of *Deutziaparviflora*, 19 October 2018, P.-L. Qiu, S.-R. Tang & L. Liu, HMJAU-PM91860 (holotype) and HMAS 248089 (isotype) in the Herbarium Mycologicum Academiae Sinica (HMAS), Beijing; ibid., on leaves of *D.parviflora*, 26 May 2018, P.-L. Qiu, S.-R. Tang & D.-N. Jin, HMJAU-PM91861 (paratype).

#### Description.

**Figure 2.**
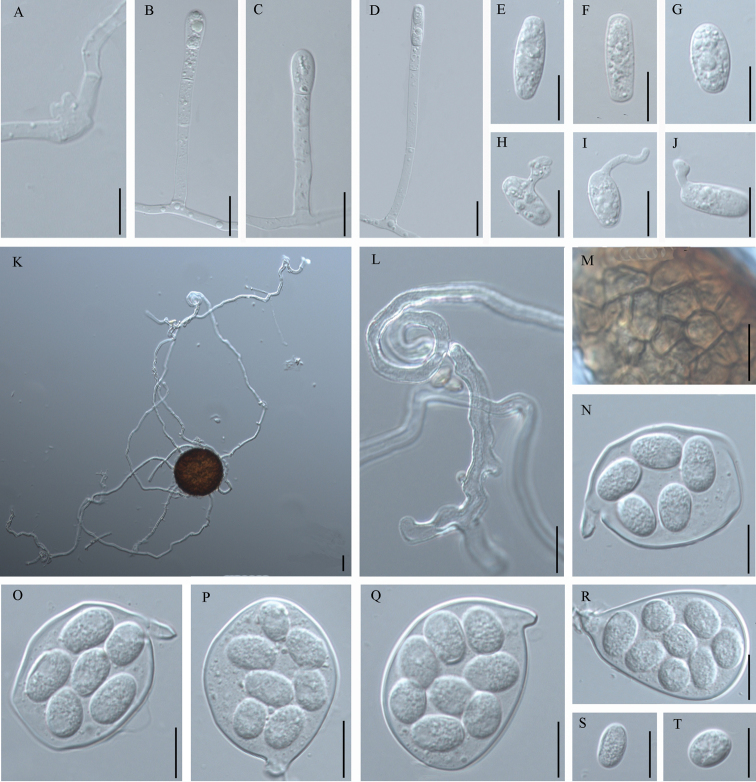
Morphology of *Erysiphedeutziicola* on *Deutziaparviflora*. **A** Lobed hyphal appresorium **B–D** Conidiophores **E–G** Conidia **H** Lobed germ tube arising from the lateral of conidium **I** Germ tube showing longitubus pattern arising from a conidium in perihilar position **J** Slightly lobed germ tube arising from the perihilar position of a conidium **K** Chasmothecium **L** Appendage with sinuous-geniculate, branched and circinate apex **M** Peridium cells **N** Ascus with 5 ascospores **O** Ascus with six ascospores **P** Ascus with seven ascospores **Q** Ellipsoid ascus with eight ascospores **R** Clavate ascus with eight ascospores **S** Ellipsoid ascospore **T** Ovoid ascospore. Scale bars: 20 μm.

Forming distinct white colonies, very small and dense, covering both sides of the leaves, causing discolourations of entire leaves or even malformations. Mycelium amphigenous, effuse and persistent. Hyphal appressoria distinctly lobed, solitary (Figure 2, A). Conidiophores, short to very long, 54.5‒171.0(‒235) × 5.8‒8.0 μm, arising from the upper surface of hyphal mother cells (Figure 2, B‒D). Foot-cells straight, (23‒)30.5‒75.0 × 5.7‒7.7 μm, followed by 1 to 3(‒4) cells, 13‒80 μm in length. Conidia formed singly, hyaline, ellipsoid-ovoid or oblong, 18.6‒35.5 × 10‒14 μm with a length/width ratio varying from 1.4‒3.0(‒3.3) (Figure 2, E‒G). Germ tubes on the conidia with lobed apex or longitubus pattern, apex simple or somewhat swollen, produced laterally, near the middle or in perihilar position (Figure 2, H‒J). Chasmothecia, amphigenous, scattered, 70‒100.0 μm diam. (Figure 2, K). Peridium cells irregularly polygonal, 3.5–12.5 µm diam. (Figure 2, M). Appendages 6‒14 per chasmothecium, mycelioid, hyaline, aseptate, extremely long and flexuous, 1.3‒7.0 times as long as the chasmothecial diameter, up to 600 μm, 3–9 µm wide in the lower half, apices mostly sinuous-geniculate or branched, circinate at the near apex, coils relatively loose and wide (Figure 2, L). Asci 4–6 per chasmothecium, broad obovoid-saccate or clavate, short-stalked or sessile, 48‒71.5 × 28.5‒49.5 μm (Figure 2, N‒R). Ascospores ovoid or ellipsoid, 5‒8 in each ascus, 13.0‒20.5 × 10.5‒14.5 μm (Figure 2, S‒T).

#### Host range and distribution.

On *Deutziaparviflora* (Hydrangeaceae) in Beijing, China.

## Discussion

For taxonomic purposes within the genus *Erysiphe*, the characteristics of the appendages are the most effective way to assign species to morphological, non-phylogenetic sections of *Erysiphe* that were introduced in Braun and Takamatsu (2000) and Braun and Cook (2012). Of the nine species recorded on hosts of the Hydrangeaceae, only *E.poeltii*pertains to Erysiphesect.Erysiphe characterised by mycelioid chasmothecial appendages. The mycelioid appendages of *E.poeltii* are unbranched and later become yellowish to brownish, but remain paler or hyaline in the upper half. The appendages of *E.deutziicola* are completely hyaline, mostly sinuous-geniculate or branched in the apical region and sometimes distinctly circinate at the apex. *Erysiphedeutziae* is currently the only species on *Deutzia* spp., but it differs from *E.deutziicola* by its typically dichotomously branched appendages. The shorter, straight, stiff uncinuloid chasmothecial appendages with uncinated-circinate tips are characteristic for *E.hydrangeae*, *E.schizophragmatis* and *E.yanshanensis* and easily distinguish these species from *E.deutziicola*. Recently published phylogenetic examinations revealed that *Pseudoidiumhortensiae* belongs to the *Erysipheaquilegiae* complex (Shin et al. 2018) suggesting that *Pse.hortensiae* is a member of sect. Erysiphe, although chasmothecia of this species have not yet been found (Braun and Cook 2012). There are two additional species with chasmothecial appendages similar to those of *E.deutziicola*, viz., *E.abeliae* R.Y. Zheng & G.Q. Chen and *E.braunii* Y. Nomura. which have been described. However, the appendages in *E.braunii* on *Saussurea* are quite distinct by being pluriseptate and not coiled at the tip and the asci are 2–3-spored (Braun and Cook 2012). *Erysiphedeutziicola* differs from *E.abeliae* in having fewer, much longer appendages (numbers 6‒14 vs. 10‒40, 1.3‒7.0 times as long as the chasmothecial diameter vs. mostly 1–2 times) and fewer asci (4‒6 per chasmothecium vs. 4‒8). In addition, the ascospores of *E.abeliae* are yellowish vs. colourless in *E.deutziicola*.

The phylogenetic analysis revealed that *Erysiphedeutziicola* clustered in a separate clade with 100% bootstrap support, distant from all included *Erysiphe* species occurring on hosts of the Hydrangeaceae and it further confirmed that this species represents a species of its own. The detail morphological traits of *E.deutziicola* and other *Erysiphe* species on hosts of the Hydrangeaceae, as well as morphologically similar species on hosts of other plant families, are shown in Table 2.

**Table 2.** Morphological comparison of *Erysiphedeutziicola* and closely related species in Braun and Cook (2012).

**Species name**	**Host family**	**Conidia (μm)**	**Length of conidiophores (μm)**	**Diameter of chasmothecia (μm)**	**Appendages**		**Number of asci**	**Ascospores**	
					**Number**	**morphology**		**Number**	**Colour**
* Erysiphe deutziicola *	Hydrangeaceae	18.6‒35.6 × 10.2‒14.1	54.7‒171.0 (‒234.7)	71.0‒100.0	6‒14	mycelioid	4‒6	5‒8	colourless
* E. abeliae *	Caprifoliaceae	‒ †	‒	(85–) 95–120	10–40	mycelioid	4–8	6–8	yellowish
* E. braunii *	Asteraceae	35–45 × 17–23	80–110	90–130	18–36	mycelioid	6–16	2–3	colourless
* E. deutziae *	Hydrangeaceae	25–35 (–40) × (16.5–) 17.5–20 (–22)	50–75	70–150	4–16	dichotomous	2–6	4–8	colourless
* E. hydrangeae *	Hydrangeaceae	‒	‒	120–225	19–40 (–48)	circinate	(5–) 6–12 (–21)	(4–) 5–8	colourless
* E. poeltii *	Hydrangeaceae	26–33 × 13–18	‒	75–110	5–20	mycelioid	(3–) 4–5 (–6)	5–8	colourless
* E. schizophragmatis *	Hydrangeaceae	27–38 × 14–18	up to 90	80–120	7–22	circinate	6–13	4–5	colourless
* E. yanshanensis *	Hydrangeaceae	average 26.5 × 14	45–80	average 120	5–23	circinate	(3–) 4–9 (–11)	(2–) 5–7 (–8)	yellowish
* Pseudoidium hortensiae *	Hydrangeaceae	(18–) 25–40 (–45) × (9–) 12–19 (–22)	40–130 (–175)	‒	‒	‒	‒	‒	‒

^†^ “‒” means no related information

## Supplementary Material

XML Treatment for Erysiphe deutziicola
